# Binuclear Rhodium(II) Complexes With Selective Antibacterial Activity

**DOI:** 10.1155/MBD.1997.81

**Published:** 1997

**Authors:** Małgorzata Bień, Tadeusz M. Lachowicz, Agnieszka Rybka, Florian P. Pruchnik, Lilianna Trynda

**Affiliations:** 1 Institute of Microbiology University of Wrocław 63/77 Przybyszewskiego Wrocław 51-148 Poland; 2 Faculty of Chemistry University of Wrocław 14 Joliot-Curie Wrocław 50-383 Poland

## Abstract

Binuclear rhodium(II) complexes [Rh_2_Cl_2_(μ-OOCR)_2_(N-N)_2_] {R = H, Me; N-N = 2,2'-bipyridine (bpy), 1,10-phenanthroline (phen)} and [Rh_2_(μ-OOCR)_2_(N-N)_2_(H_2_O)_2_](RCOO)_2_ (R = Me, Et;) have been synthesized and their structure and properties have been studied by electronic, IR and ^1^H NMR spectroscopy. Antibacterial activity of these complexes against *Escherichia coli* and *Staphylococcus aureus* has been investigated. The most active antibacterial agents against *E. coli* were [Rh_2_Cl_2_(μ-OOCR)_2_(N-N)_2_] and [Rh_2_(μ-OOCR)_2_(N-N)_2_(H_2_O)_2_](RCOO)_2_ {R = H and Me} which were considerably more active than the appropriate nitrogen ligands. The complexes show low activity against *S. aureus*. The activity of the complexes [Rh_2_(OOCR)_2_(N-N)_2_(H_2_O)_2_](OOCR)_2_ against *E. coli* decreases in the series: R=H≅CH_3_>C_2_H_5_>C_3_H_7_≅C_4_H_9_. The reverse order was found in the case of *S. aureus*.


### BINUCLEAR RHODIUM(II) COMPLEXES WITH SELECTIVE ANTIBACTERIAL ACTIVITY

Małgorzata Bień[1], Tadeusz M. Lachowicz[1], Agnieszka Rybka[1], Florian P. Pruchnik*[2], and Lilianna Trynda[2]

[1] Institute of Microbiology, University of Wrocław, 63/77 Przybyszewskiego, 51-148 Wrocław, Poland

[2] Faculty of Chemistry, University of Wrocław, 14 Joliot-Curie, 50-383 Wrocław, Poland

**Abstract**
Binuclear rhodium(II) complexes $[Rh_2Cl_2(\mu\text{-}OOCR)_2(N\text{-}N)_2]$ {R = H, Me; N-N = 2,2'-bipyridine (bpy), 1,10-phenanthroline (phen)} and $[Rh_2(\mu\text{-}OOCR)_2(N\text{-}N)_2(H_2O)_2](RCOO)_2$ (R = Me, Et;) have been synthesized and their structure and properties have been studied by electronic, IR and $^1H$ NMR spectroscopy. Antibacterial activity of these complexes against *Escherichia coli* and *Staphylococcus aureus* has been investigated. The most active antibacterial agents against *E. coli* were $[Rh_2Cl_2(\mu\text{-}OOCR)_2(N\text{-}N)_2]$ and $[Rh_2(\mu\text{-}OOCR)_2(N\text{-}N)_2(H_2O)_2](RCOO)_2$ {R = H and Me} which were considerably more active than the appropriate nitrogen ligands. The complexes show low activity against *S. aureus*. The activity of the complexes $[Rh_2(OOCR)_2(N\text{-}N)_2(H_2O)_2](OOCR)_2$ against *E. coli* decreases in the series: R = H ≅ $CH_3$ > $C_2H_5$ > $C_3H_7$ ≅ $C_4H_9$. The reverse order was found in the case of *S. aureus*.

**Introduction**

Binuclear rhodium(II) tetracarboxylato complexes $[Rh_2(\mu\text{-}OOCR)_4L_2]$ as well as complexes containing two bridging ligands $[Rh_2X_2(\mu\text{-}OOCR)_2(N\text{-}N)_2]$ and $[Rh_2(\mu\text{-}OOCR)_2(N\text{-}N)_2L_2]^{2+}$, where L = Lewis base, X = halide and N-N = 2,2'-bipyridine, 1,10-phenanthroline and their derivatives have aroused considerable interest because of interesting structure and reactivity [1-9], catalytic properties [1 - 4, 10] and anticancer activity [1 - 4, 11 - 17]. The antitumor activity of $Rh_2(OOCR)_4$ complexes intensified studies of their interaction with biomolecules, e.g. aminoacids, peptides, vitamins, nucleic acid bases, adenosine phosphates, DNA, etc. [1 - 4, 12, 14 - 21]. Tetraacetatodirhodium(II) [20] and $[Rh_2(OOCCH_3)_2(bpy)_2(H_2O)_2](OOCCH_3)_2$ [21] bind effectively to human serum albumin via imidazole rings leading to the conformational changes of HSA.

It has been found [22] that $[Rh_2(OOCR)_2(N\text{-}N)_2(H_2O)_2]^{2+}$ complexes show cytostatic activity for human oral carcinoma KB cell line in vitro. Some of the determined biological activities were in close relation with findings obtained on synchronous cultures of green algae exposed to these Rh(II) complexes [23]. New metal compounds possessing antimicrobial activity are recently intensively investigated because of increasing resistance of microbes against many antibiotics. It has been found that some platinum complexes possessing antitumor activity show also antibacterial properties. A further well defined series of metal complexes with antibacterial activity is that of rhodium(III) coordination compounds of formula *trans*-$[RhX_2(py)_4]Y$ [24 - 26]. These complexes are more active against Gram-positive microorganisms than Gram-negative ones but where Gram-negative bacteria, such as *E. coli*, are susceptible the inhibitory concentrations are approximately the same. The most lipophilic rhodium complexes are the most bacteriostatic, this perhaps correlating with penetration. However, we have found that some dimeric rhodium(II) complexes $[Rh_2(OOCR)_2(N\text{-}N)_2(H_2O)_2]^{2+}$ (N-N = bpy, phen, 6,7-dimethyl-2,3-bis(2-pyridyl)quinoxaline, R = $CH_2CH_2CH_3$, $CH_2(CH_2)_2CH_3$, CH(OH)Ph, Ph) are considerably more active against Gram-positive than against Gram-negative bacteria [27]. The complexes $[RhCl_2(bpy)_2]^+$ are not active antibacterial agents, perhaps because they possess the *cis*-configuration [24 - 26]. . In this paper, we report on synthesis and properties of dimeric rhodium(II) complexes $[Rh_2(OOCR)_2(N\text{-}N)_2(H_2O)_2]^{2+}$ and $[Rh_2Cl_2(OOCR)_2(N\text{-}N)_2]$ (R = H, $CH_3$, $CH_2CH_3$, N-N = 2,2'-bipyridine, 1,10-phenanthroline) which are much more active against Gram-negative than against Gram-positive bacteria.

**Experimental**

*Synthesis of the complexes.*

Complexes $[Rh_2(OOCCH_3)_4(H_2O)_2]$ [28], $Na_4[Rh_2(CO_3)_4]\cdot 2.5H_2O$ [29], $[Rh_2(OOCCH_3)_2(phen)_2(H_2O)_2](OOCCH_3)_2$ (**1**) [23], $[Rh_2Cl_2(OOCH)_2(phen)_2]$ (**2**) [5, 7], $[Rh_2Cl_2(OOCCH_3)_2(phen)_2]$ (**3**) [22], $[Rh_2Cl_2(OOCCH_3)_2(bpy)_2]$ (**5**) [22], $[Rh_2(OOCCH_3)_2(dmpq)_2(H_2O)_2](OOCCH_3)_2$ (**9**) [27] and $[Rh_2(OOCPh)_2(phen)_2\text{-}(H_2O)_2](OOCPh)_2$ (**10**) [27], $[Rh_2(OOCCH_2CH_2CH_3)_2(bpy)_2(H_2O)_2](OOCCH_2CH_2CH_3)_2$ (**11**) [27], $[Rh_2\{OOC(CH_2)_3CH_3\}_2(phen)_2(H_2O)_2]\{OOC(CH_2)_3CH_3\}_2$ (**12**) [27] and $[Rh_2\{OOC(CH_2)_3CH_3\}_2(bpy)_2(H_2O)_2]\{OOC(CH_2)_3CH_3\}_2$ (**13**) [27] were prepared by literature methods.

1,10-Phenanthroline (phen), 6,7-dimethyl-2,3-bis(2-pyridyl)quinoxaline (dmpq) and 2,2'-bipyridine (bpy) were obtained from Aldrich and used without further purification.

**$[Rh_2(OOCCH_3)_2(bpy)_2(H_2O)_2](OOCCH_3)_2$ (4).** A solution of $[Rh_2(OOCCH_3)_4(H_2O)_2]$ (0.239 g, 0.5 mmol) and 2,2'-bipyridine (0.156 g, 1 mmol) in 5 $cm^3$ of dioxane was heated at reflux for 3 h, cooled to the room temperature and brown precipitate has been filtered out. The product has been dissolved in ethanol (2 $cm^3$) and pure dark red complex **4** has been deposited by means of diethyl ether. Precipitate was filtered off, washed with $(C_2H_5)_2O$ and dried under reduced presure, yield 0.296 g, 75%. Anal. Calc. for $C_{28}H_{32}N_4O_{10}Rh_2$: C 42.55, H 4.08, N 7.09. Found: C 42.16, H 4.25, N 6.90.

**$[Rh_2(OOCCH_2CH_3)_2(phen)_2(H_2O)_2](OOCCH_2CH_3)_2 \cdot 2H_2O$ (6).** A solution of $[Rh_2(OOCCH_2CH_3)_4(H_2O)_2]$ (0.1335 g, 0.25 mmol, ) and phen (0.099 g, 0.5 mmol) was heated in ethanol (7 $cm^3$) for 2 h, cooled to the room temperature and filtered in atmosphere of air. The brown-red precipitate was deposited by means of diethyl ether. The product was washed with ether and dried in vacuo, yield 0.159 g, 71%. Yield was approximately the same when 1,4-dioxane or isopropanol were used as a solvent. Anal. Calc. for: $C_{36}H_{44}N_4O_{12}Rh_2$:: C 46.47, H 4.77, N 6,02. Found: C 46.10, H 4.82, N 5.96.

**$[Rh_2(OOCCH_2CH_3)_2(bpy)_2(H_2O)_2](OOCCH_2CH_3)_2$ (7).** A solution of $[Rh_2(OOCCH_2CH_3)_4(H_2O)_2]$ (0.1335 g, 0.25 mmol, ) and bpy (0.078 g, 0.5 mmol) was heated in ethanol ( 10 $cm^3$) for 2 h. The solution was evaporated to dryness and compound was recrystallized from *iso*-propanol-*tert*-butanol (1 : 2) The red precipitate was washed with diethyl ether and dried in vacuo, yield 0.134 g, 63%. Isopropanol and 1,4-dioxane may be also used as solvents instead of ethanol. Anal. Calc. for $C_{32}H_{40}N_4O_{10}Rh_2$: C 45.40, H 4.76, N 6.62. Found: C 45.70, H 4.68, N 6.70.

**$[Rh_2(OOCCH_2CH_3)_4(H_2O)_2]$. (8).** A mixture of $Na_4[Rh_2(CO_3)_4] \cdot 2.5H_2O$ (0.583 g, 1 mmol) and propionic acid (0.593 g, 8 mmol) in water ( 10 $cm^3$) was heated at 100 °C for 2 h. The green product was collected and washed with water and methanol and dried in vacuo, yield 0.411 g, 0,77%. Anal. Calc. for $C_{12}H_{24}O_{10}Rh_2$: C 28.94, H 4.05. Found: C 28.70, H 3.96.

*Physical measurements.*

Infrared spectra (KBr pellets and nujol mulls) were measured on a Perkin Elmer 283 and a Bruker IFS 113v, UV-VIS on a Cary 5 and Beckman DU 7500, and $^1H$ NMR spectra on a Bruker AMX 300.

*Antibacterial activity.*

The antibacterial activity was evalueted on reference strains *Staphylococcus aureus* 209P (Oxford) and *Escherichia coli* K-12 ROW. Nutrient broth and nutrient agar (Biomed, Warszawa) as growth and plating media were used, respectively. The media were supplemented with the tested compounds at suitable concentrations. The growth of bacteria was determined after 24 hours incubation at 37 °C. Antibacterial activity was determined either as MIC (minimal inhibitory concentration) (in solid medium) or by serial dilution in nutrient broth by the method presented in the previous paper [27]. In the former about one hundred colony forming units per plate were plated; in the latter 10 $cm^3$ of broth was inoculated with either $10^8$ or $10^5$ colony forming units.

In experiments on mutagenicity of the complexes the test recommended by Ames and Maron [30] was carried out using TA 97, TA 98, TA 100 and TA 102 as bio⁻his⁻ mutants of *Salmonella typhimurium.*

### Results and Discussion

The $[Rh_2(RCOO)_2(N\text{-}N)_2(H_2O)_2][RCOO]_2$ complexes were prepared by reactions between $[Rh_2(RCOO)_4(H_2O)_2]$ and appropriate nitrogen ligand in ethanol, 2-propanol and 1,4-dioxane. Other alcohols may be also used. The reactions in primary and secondary alcohols should be performed in the presence of air because rhodium(II) complexes during heating in these solvents are reduced to the deep green or green-blue rhodium(I) compounds which are very easily reoxidized to Rh(II) complexes with oxygen or air. Thus, the $[Rh_2(RCOO)_2(N\text{-}N)_2(H_2O)_2][RCOO]_2$ complexes can be used for oxidation of alcohols to carboxylic acid. The propionato complexes during prolongated heating in ethanol in the presence of air can be partly converted into appropriate acetates because of oxidation of $C_2H_5OH$. Therefore syntheses of the $[Rh_2(RCOO)_2(N\text{-}N)_2(H_2O)_2][RCOO]_2$ and $[Rh_2Cl_2(RCOO)_2(N\text{-}N)_2]$ complexes in alcohols should be carried out in neutral atmosphere and rhodium(I) compounds formed after heating of the reaction mixture should be immadiately cooled and oxidized to the binuclear Rh(II) complexes with air and deposited. The $[Rh_2Cl_2(RCOO)_2(N\text{-}N)_2]$ complexes can be prepared analogously in the presence of stoichiometric amounts of NaCl [5, 7, 22]. The $[Rh_2(RCOO)_2(N\text{-}N)_2(H_2O)_2][RCOO]_2$ and $[Rh_2Cl_2(RCOO)_2(N\text{-}N)_2]$ compounds are stable under air atmosphere both in solid state and in solutions. The $[Rh_2(RCOO)_2(N\text{-}N)_2(H_2O)_2][RCOO]_2$ complexes are readily soluble in water, methanol and ethanol and slightly soluble in higher alcohols, insoluble in diethyl ether, 1,4 dioxane and nonpolar solvents. Solubility of the $[Rh_2Cl_2(RCOO)_2(N\text{-}N)_2]$ compounds in the same solvents is lower. All complexes have been characterized by a combination of elemental analysis and IR, UV-VIS and $^1H$ NMR spectroscopy. The data are collected in Tables 1-3.

The $[Rh_2(RCOO)_2(N\text{-}N)_2(H_2O)_2][RCOO]_2$ compounds as well as chloro complexes $[Rh_2Cl_2(OOCR)_2(N\text{-}N)_2]$ in solutions in methanol give conductivities in the 100 - 150 $ohm^{-1}.cm^2.mol^{-1}$ range. Thus $[Rh_2Cl_2(OOCR)_2(N\text{-}N)_2]$ complexes show incomplete substitution of chloro for $H_2O$ or $CH_3OH$ ligands and in solution concentration of $[Rh_2Cl(OOCR)_2(phen)_2(H_2O)]^+$ ions is relatively high. This was also confirmed by the presence of ClØRh CT bands in the electronic spectra of these compounds in the 290 - 255 nm [4, 5, 31].

In the electronic spectra (Table 1) of these complexes in visible region two bands are observed. Band I occurs at about 555 -570 nm, about 20 - 30 nm lower compared with that for $[Rh_2(RCOO)_4(H_2O)_2]$ compounds because this band corresponds to the $\pi^*(Rh_2)$Ø$\sigma^*(Rh_2)$ transition [1 - 5, 31] and the $\pi^*(Rh_2)$ levels in $[Rh_2(RCOO)_2(N\text{-}N)_2(H_2O)_2]^{2+}$ complexes due to the interaction of the orbitals of the $Rh_2$ core with $\pi^*$ orbitals of nitrogen ligands have lower energy than that of the $\pi^*$ $(Rh_2)(5e_g)$ orbitals in $[Rh_2(OOCR)_4(H_2O)_2]$ compounds. The intense band II at about 408 - 420 nm was assigned to the allowed charge-transfer transition $\sigma(Rh_2)$Ø$\pi^*(N\text{-}N)$ [31]. This band obscures the absorption of low intensity attributed to the $\pi^*(Rh_2)$Ø$\sigma^*(Rh\text{-}O)$ transition observed at 430 - 460 nm in $[Rh_2(OOCR)_4(H_2O)_2]$ complexes. The energy of the band I strongly depends on the nature of axial ligands and encreases with increasing of the field strength of these ligands. Band I remains relatively constant for axial oxygen donor ligands, but is blue shifted with nitrogen donor ligand.

Table 1. Electronic Spectra of Rhodium(II) Complexes.

| Compound | Band, $\lambda(\varepsilon)$, [nm]($M^{-1}cm^{-1}$) |
|---|---|
| **3** in $H_2O$ | 562(200), 408(2550), 366sh(3850), 334sh(5200), 268(29200), 252(30800), 233(19000) |
| **4** in $H_2O$ | 564(340), 416(2400), 362(3700), 305(14800), 270(30200), 257(32400) |
| **5** in $H_2O$ | 559(270), 416(2600), 356(3800), 325sh(8200), 303sh(16800), 268(31200), 255(31200), 227sh(12800) |
| **6** in $H_2O$ | 560(290), 411(2500)sh, 360(4300)sh, 332(5100)sh, 271(31600), 253(39300), 224(46300) |
| **7** in $H_2O$ | 560(380), 420(2300), 361(3500), 304(15100), 270(29600)sh, 259(31800), |

Table 2. IR Spectra of Rhodium(II) Complexes

| Complex | $\nu$(CH) [$cm^{-1}$] | $\nu^{as}$(COO) [$cm^{-1}$] | $\nu^{s}$(COO) [$cm^{-1}$] | other bands [$cm^{-1}$] |
|---|---|---|---|---|
| **1** | 3090vw, 3058w, 3000vw, 2925vw | 1595vs, 1582vs, 1560vs | 1445sh, 1430vs, 1412vs | 1520m, 1494w, 1360s, 1323s, 1222w, 1205w, 1147w, 1112vw, 1090vw, 1048w, 1020w, 841s, 766w, 724sh, 715s, 703s, 648m, 622w, 516w, 480vw, 440vw |
| **4** | 3115w, 3080w, 3040vw, 2990vw, 2930vw | 1602m, 1578vs, 1560vs | 1445vs, 1418vs, 1400vs | 1488w, 1464m, 1350w, 1332w, 1305w, 1268vw, 1243w, 1152w, 1120vw, 1102vw, 1062vw, 1040w, 1012w, 914w, 762s, 718m, 699s, 642m, 620w, 460vw, 415w |
| **6** | 3060w, 3010vw, 2985m, 2930w, 2920w, 2880vw | 1594s, 1565sh, 1550vs | 1460s, 1425vs, 1405vs, 1372s, 1360s | 1510w, 1490vw, 1337w, 1294m, 1276m, 1222w, 1205w, 1142w, 1110vw, 1088vw, 1070m, 1033w, 1010w, 892m, 878w, 867w, 850sh, 836s, 822vw, 807w, 767vw, 742vw, 710s, 678vw, 650vw, 610vw, 510vw, 433vw, 408vw, 386vw, 372vw, 324vw |
| **7** | 3115w, 3075m, 3060w, 3025w, 2985m, 2960w, 2940sh, 2925m, 2875w | 1600sh, 1562vs, 1550vs | 1463s, 1446s, 1320s, 1380m, 1362m | 1490vw, 1305s, 1280s, 1246w, 1170m, 1162m, 1121vw, 1110w, 1073m, 1042vw, 1012w, 1001vw, 898m, 863m, 808m, 765vs, 720s, 699w, 678vw, 644w, 612w, 515w br, 418m,.408sh, 375vw, 345vw, 328vw |
| **8** | 2987m, 2980m, 2945m, 2920w, 2880 | 1568vs | 1470s, 1462s, 1450m, 1420vs | 1382m, 1370s, 1299vs, 1242m, 1187m, 1179m, 1170m, 890m, 804m, 745w, 710m, 677s, 632vw, 432m, 410m, 352s |

The IR spectra of complexes with phenanthrolines confirmed that carboxylato ligands are symmetrically bonded to the both rhodium atoms because differences between $\nu^{as}(COO)$ and $\nu^{s}(COO)$ are small (Table 2).

The chemical shifts of protons of carboxylato ligands in $[Rh_2(RCOO)_2(N\text{-}N)_2(H_2O)_2]^{2+}$ are higher than those of non-coordinated carboxylates and RCOO groups in $Rh_2(OOCR)_4(H_2O)_2$ complexes. The highest coordination shift is observed for protons bound with corboxylic group.The Rh - H coupling was observed only in the case of formato complexes for which $^3J_{RhH}$ is *ca* 4 Hz. The 1,10-phenanthroline and 2,2'-bipyridine ligands in $[Rh_2(OOCR)_2(phen)_2(H_2O)_2](OOCR)_2$, $[Rh_2(OOCR)_2(bpy)_2(H_2O)_2](OOCR)_2$, $[Rh_2Cl_2(OOCR)_2(phen)_2]$ and $[Rh_2Cl_2(OOCR)_2(bpy)_2]$ (R = H, alkyl) are symmetrically bound to the rhodium atoms. This is proven by the presence only 4 signals of hydrogen atoms of heterocyclic nitrogen ligands in the $^1H$ NMR spectra of these complexes (Table 3).

Table 3. $^1H$ NMR Spectra of Rhodium(II) Complexes.

| Compound | δ, ppm (J, Hz) |
|---|---|
| **1** in $D_2O$ | 1.849s($CH_3^{nc}$), 2.624s($CH_3^c$), 7.558dd($H^3$,$J_{23}$=5.3 Hz, $J_{34}$=8.0 Hz), 7.658s($H^5$), 8.224dd($H^4$, $J_{24}$ = 1.1 Hz), 8.455d($H^2$) |
| **2** in $D_2O$ | 7.464 dd($H^3$, $J_{23}$ = 5.3 Hz, $J_{34}$ = 8.3 Hz), 7.557s($H^5$), 8.127d($H^4$), 8.355t(HCOO, $J_{RhH}$ = 4.1Hz), 8.419d($H^2$) |
| **3** in $D_2O$ | 2.536s($CH_3$), 7.452dd($H^3$, $J_{23}$ = 5.4 Hz, $J_{34}$ = 8.3 Hz), 7.551s($H^5$), 8.113dd($H^4$, $J_{24}$ = 0.8 Hz), 8.348d($H^2$) |
| **4** in $D_2O$ | 1.804s($CH_3^{nc}$), 2.456s($CH_3^c$), 7.290ddd($H^5$,$J_{56}$=5.8 Hz, $J_{45}$=7.0 Hz, $J_{35}$= 1.8 Hz ), 7.77-7.86m ($H^3$+$H^4$), 8.203d($H^6$) |
| **5** in $D_2O$ | 2.418s($CH_3$), 7.249ddd($H^5$, $J_{56}$ = 5.6 Hz, $J_{45}$ = 7.3 Hz), 7.729 - 7.811m($H^3$+$H^4$), 8.159d($H^6$) |
| **6** in $D_2O$ | 0.932t($CH_3^{nc}$, $^3J_{HH}$ = 7.7 Hz), 1.339t($CH_3^c$, $^3J_{HH}$ = 7.6 Hz), 2.056q($CH_2^{nc}$, $^3J_{HH}$ = 7.7 Hz), 2.823q($CH_2^c$, $^3J_{HH}$ = 7.6 Hz), 7.487dd($H^3$,$J_{23}$ = 5.3 Hz, $J_{34}$ = 8.3 Hz), 7.594s($H^5$), 8.156dd($H^4$, $J_{24}$= 1.0 Hz), 8.351d($H^2$) |
| **7** in $D_2O$ | 0.933t($CH_3^{nc}$, $^3J_{HH}$ = 7.7Hz), 1.244t($CH_3^c$, $^3J_{HH}$ = 7.6 Hz), 2.058q($CH_2^{nc}$, $^3J_{HH}$ = 7.7 Hz), 2.699q($CH_2^c$, $^3J_{HH}$ = 7.6 Hz), 7.268ddd($H^5$, $J_{45}$=7.0, $J_{56}$=5.7 Hz), 7.75-7.84m($H^3$+$H^4$), 8.146d($H^6$) |
| **8** in $D_2O$ | 1.114t(12H, $CH_3$, $^3J_{HH}$ = 7.6 Hz), 2.272q(8H, $CH_2$) |

nc - noncoordinating ion, c - coordinating ligand, m - complex pattern

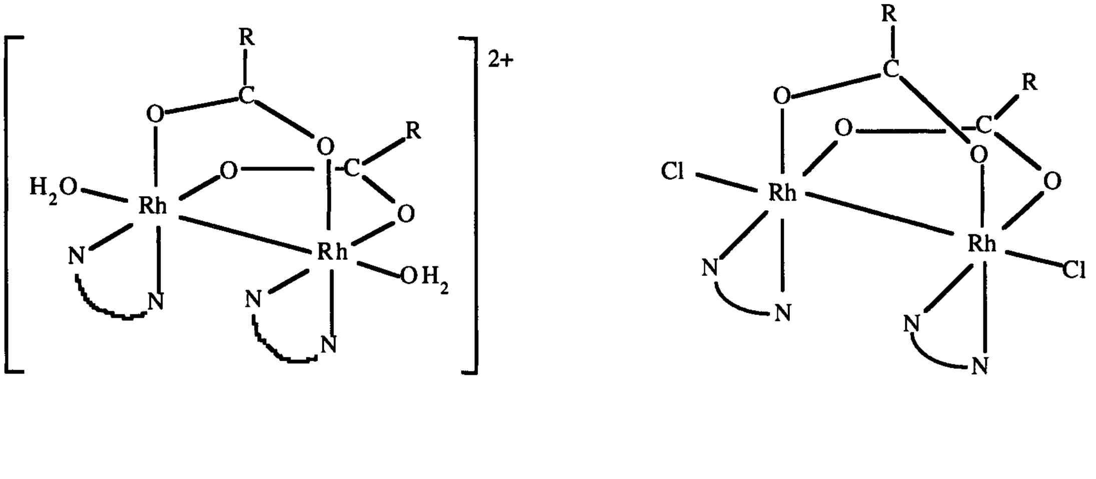

Fig. 1. Structure of $[Rh_2(OOCR)_2(N\text{-}N)_2(H_2O)_2]^{2+}$ and $[Rh_2Cl_2(OOCR)_2(N\text{-}N)_2]$ complexes

Thus, the physicochemical measurements indicate that structure of $[Rh_2(OOCR)_2(N\text{-}N)_2(H_2O)_2](OOCR)_2$ complexes is analogous to that of $[Rh_2Cl_2(RCOO)_2(N\text{-}N)_2]$ [4, 5, 7] with symmetric carboxylato bridges and water molecules coordinating along Rh-Rh axis (Fig. 1).

The complexes **1** - **7** and the ligands (phen and bpy) were tested *in vitro* for antibacterial activity against Gram-negative *Escherichia coli* ROW and Gram-positive *Staphylococcus aureus* 209 P (Oxford) strains (Table 4). The highest activity against Gram-negative bacteria exhibited complexes **1** - **5** and compounds **6** and **7** are a little less active, however, they are considerably more active than the appropriate nitrogen ligands. They are active at concentrations $5.10^{-6}$ - $10^{-5}$ M. All complexes (**1** - **7**) show rather low activity against *S. aureus.*

For some $[Rh_2(OOCR)_2(N\text{-}N)_2(H_2O)_2](OOCR)_2$ and $[Rh_2Cl_2(RCOO)_2(N\text{-}N)_2]$ complexes characteristic is high selectivity against Gram-negative or Gram-positive bacteria. In general, Gram-positive microorganisms are more susceptible than gram-negative ones, the minimal inhibitory concentrations are usually ten times lower for the former ($10^{-6}$ M) than for the latter ($10^{-5}$ M). The activity of the complexes $[Rh_2(OOCR)_2(N\text{-}N)_2(H_2O)_2]\text{-}(OOCR)_2$ against *E. coli* decreases in the series: R = H ≅ $CH_3$ > $C_2H_5$ > $C_3H_7$ ≅ $C_4H_9$. The reverse order was found in the case of *S. aureus*. Thus, $[Rh_2(OOCR)_2(N\text{-}N)_2\text{-}(H_2O)_2](OOCR)_2$ complexes with the most hydrophobic R group are the most active against *S. aureus* [27], this presumably correlating with penetration. This was also confirmed for analogous complexes containing PhCOO and PhCH(OH)COO ligands [27]. The data given in the Table 4 indicate that the most hydrophilic $[Rh_2(OOCR)_2(N\text{-}N)_2(H_2O)_2](OOCR)_2$ complexes are the most active against *E. coli*.

Table 4. Viable count of *E.coli* K-12 ROW and *S. aureus* P-209 OXFORD in the presence of binuclear rhodium complexes in solid agar medium

| Complex No | concentration, M | Percent of c.f.c. *E.coli* | *S. aureus* | Complex No | concentration, M | Percent of c.f.c. *E.coli* | *S. aureus* |
|---|---|---|---|---|---|---|---|
| **1** | $1.10^{-5}$<br>$5.10^{-6}$<br>$2.5.10^{-6}$ | 0<br>0<br>80 | 100<br>-<br>- | **7** | $1.10^{-5}$ | 4.0 | 40 |
| **2** | $1.10^{-5}$<br>$2.10^{-6}$ | 0<br>80 | 90<br>100 | **9** | $1.10^{-5}$<br>$2.5.10^{-6}$ | 100<br>- | 0<br>0 |
| **3** | $1.10^{-5}$<br>$2.10^{-6}$ | 0<br>82 | 66<br>100 | **10** | $1.10^{-5}$<br>$1.25.10^{-6}$ | 100<br>- | 0<br>2.5 |
| **4** | $1.10^{-5}$ | 0 | 90 | **11** | $1.10^{-5}$ | 100 | 0 |
| **5** | $1.10^{-5}$ | 0 | 95 | **12** | $1.10^{-5}$<br>$5.10^{-6}$ | 100<br>- | 0<br>0 |
| **6** | $1.10^{-5}$ | 0.9 | 100 | **13** | $1.10^{-5}$<br>$5.10^{-6}$ | 100<br>- | 0<br>0 |
| phen | $1.10^{-5}$ | 37 | 21 | bpy | $1.10^{-4}$ | 74 | 34 |
| control | 0 | 100 | 100 | dmpq | $1.10^{-4}$ | 60 | 33 |

In order to get more information about the nature of the inhibitory activity of the investigated compounds, we have examined the dependence of survival of *E. coli* in nutrient broth containing complexes **1** and **4** at 37 $^0$C on time (Fig. 2).

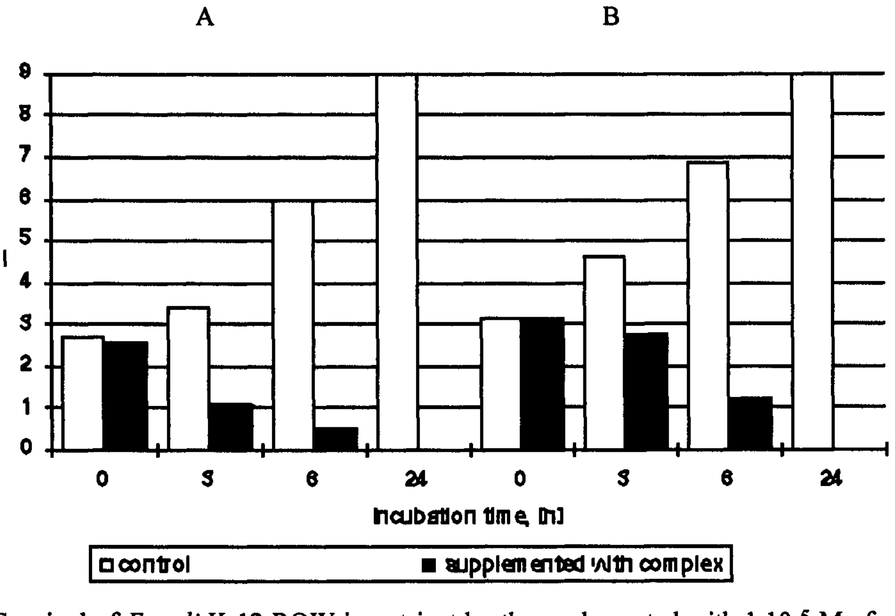

Fig. 2. Survival of *E. coli* K-12 ROW in nutrient broth supplemented with $1.10^{-5}$ M of complex **1** (A) and complex **4** (B).

It is known that some antibacterial drugs, like β-lactamase are active only against growing bacteria [32]. The fast decrease of c.f.c. (colony forming cells) with time indicates that the complexes show bactericidal activity against *E. coli* K-12 ROW. The bactericidal activity was also confirmed by analogous tests performed in saline containing complexes **1** and **4** ($10^{-5}$ M) at 37 $^0$C. The results given in Table 5 indicate that in these experiments the decrease of survival is also observed, although this decrease is not so strong as in the case of broth cultures.

The effectivity of many antibiotics and chemoterapeutic agents depends on the size of the population of treated microorganisms. Moreover, the probability of growth of the mutated cells increases with inoculum size. Therefore, the survival of *E. coli* cultures in broth over the population range $10^5$ to $10^8$ cells in the presence of complexes **1 - 7** has been determined. The results presented in Table 6 indicate that the bactericidal activity of these compounds depends on the inoculum size. The complexes **1, 3** and **5 - 7** are active at concentration equal to or greater than $5.10^{-5}$ M in broth inoculated with $10^8$ cells, while **2** is effective at $2.5.10^{-5}$ M. The lowest activity, at this inoculum size, shows complex **4**. The complexes **1 - 7** inhibited the growth of *E. coli* in broth at concentration at least for one order lower when the inoculum size was $10^5$ cells.

Table 5. Survival of *E.coli* K-12 ROW in saline supplemented with the complexes **1** and **4**

| Time of incubation (h) | Saline | | | | | |
|---|---|---|---|---|---|---|
| | Unsupplemented | | Supplemented with 1 x $10^{-5}$ M of complex | | | |
| | | | complex **1** | | complex **4** | |
| | c.f.c./cm$^3$ | % of survival | c.f.c./cm$^3$ | % of survival | c.f.c./cm$^3$ | % of survival |
| 0 | $1,3.10^3$ | 100 | $2,8.10^2$ | 100 | $1,4.10^3$ | 100 |
| 3 | $9,6.10^2$ | 74 | $1,2.10^2$ | 43 | $1,1.10^3$ | 79 |
| 6 | $1,0.10^3$ | 77 | $1,5.10^2$ | 54 | $9,5.10^2$ | 68 |
| 24 | $9,2.10^2$ | 71 | $6,0.10^1$ | 21 | $3,4.10^2$ | 24 |

c.f.c. = colony forming cells

The $[Rh_2(OOCR)_2(N\text{-}N)_2(H_2O)_2](OOCR)_2$ and $[Rh_2Cl_2(RCOO)_2(N\text{-}N)_2]$ complexes belong to coordination compounds showing rather high antibacterial activity against *E. coli* and particularly against *S. aureus* [27] and they can be considered as the antibacterial agents with possible application. Therefore, it seemed interesting to investigate the mutagenicity some of these compounds. The performed tests of Ames (Table 7) indicate that the number of the $his^+bio^+$ revertants of *Salmonella typhimurium* strains on plates containing complexes **1 - 7, 8** and **10** at concentration equal to MIC is not greater than that on control plates.

Table 6. Dependence of growth of *E.coli* K-12 ROW in broth supplemented with rhodium(II) complexes on inoculum size.

| Complex | Inoculum size | Concentration of the complex, (M) | | | | | | | |
|---|---|---|---|---|---|---|---|---|---|
| | | $1.10^{-4}$ | $5.10^{-5}$ | $2,5.10^{-5}$ | $1,25.10^{-5}$ | $6,25.10^{-6}$ | $3,125.10^{-6}$ | $1,56.10^{-6}$ | 0 |
| **1** | $10^8$ | - | -/+ | -/+ | + | + | + | + | + |
| **2** | | - | - | - | + | + | + | + | + |
| **3** | | - | -/+ | -/+ | + | + | + | + | + |
| **4** | | - | + | + | + | + | + | + | + |
| **5** | | - | -/+ | + | + | + | + | + | + |
| **6** | | - | -/+ | + | + | + | + | + | + |
| **7** | | - | -/+ | + | + | + | + | + | + |
| **1** | $10^5$ | - | - | - | - | - | - | + | + |
| **2** | | - | - | - | - | -/+ | + | + | + |
| **3** | | - | - | - | - | - | - | + | + |
| **4** | | - | - | - | - | - | + | + | + |
| **5** | | - | - | - | - | - | - | + | + |
| **6** | | - | - | - | - | + | + | + | + |
| **7** | | - | - | - | - | + | + | + | + |

+ growth as in control; - no growth -/+ growth scarcely visible

Table 7. Test of Ames on mutagenicity of rhodium(II) complexes.

| Complex | Concentration of complex | Mean number of his+bio+ revertants in the strains *S. typhimurium* | | | |
|---|---|---|---|---|---|
| | (M) | TA97 | TA98 | TA100 | TA102 |
| **1** | $1.10^{-5}$ | 183 | 45 | 56 | 364 |
| **2** | $1.10^{-5}$ | 186 | 49 | 43 | 326 |
| **3** | $5.10^{-5}$ | 97 | 12 | 33 | 326 |
| **4** | $1.10^{-5}$ | 199 | 53 | 44 | 462 |
| **5** | $1.10^{-5}$ | 115 | 48 | 58 | 293 |
| **6** | $1.10^{-5}$ | 187 | 52 | 56 | 446 |
| **7** | $1.10^{-5}$ | 192 | 43 | 58 | 449 |
| **9** | $5.10^{-6}$ | 232 | 49 | 49 | 442 |
| **10** | $2,5.10^{-6}$ | 215 | 41 | 66 | 491 |
| control | 0 | 238 | 54 | 71 | 665 |

The lower number of revertants in the presence of complexes are to be expected because at MIC concentration of complexes some of the treated cells are killed. It is difficult to determine the fraction of survivals in these experiments because it depends on inoculum size. Nevertheless, the tested complexes seem to be rather safe from the point of view of mutagenicity.

**Acknowledgements**
The authors are grateful to the KBN for support of this work (grant No. 2 P303 02005).

**References:**

[1] F.A. Cotton, R.A.Walton, *Multiple Bonds Between Metal Atoms*, Clarendon Press, Oxford, 1993;
[2] E.B. Boyar, S.D. Robinson, *Coord. Chem. Rev.*, **50**, 109(1983);
[3] T.R. Felthouse, *Progr. Inorg. Chem.*, **29**, 73(1982).
[4] F. Pruchnik, *Pure Appl. Chem.*, **61**, 795(1989);
[5] F. Pruchnik, B.R. James, P. Kvintovics, *Can. J. Chem.*, **64**, 936(1986); F. Pruchnik, M. Zuber, H. Pasternak, K. Wajda, *Spectrochim. Acta*, **34A**, 1111(1978); F.P. Pruchnik, J. Hanuza, K. Hermanowicz, K. Wajda-Hermanowicz, M. Zuber, ibid.;**45A**, 835(1989); F. Pruchnik, M. Zuber, *Rocz. Chem.*, **51**, 1813(1977);
[6] F. Pruchnik, A. Jezierski, E. Kalecińska, *Polyhedron*, **10**, 2551(1991);
[7] H. Pasternak, F. Pruchnik, *Inorg. Nucl. Chem. Letters*, **12**, 591(1976); T. Głowiak, F.P. Pruchnik ; M. Zuber, *Polish J. Chem.*, **65**, 1749(1991);
[8] A. Szymaszek, F.P. Pruchnik, *Polish J. Chem.*, **66**, 1859(1992).
[9] J. Halpern, E. Kimura, J. Molin-Case, C.S. Wong, *J. Chem. Soc., Chem. Commun.*, 1207(1971).
[10] H. Pasternak, E. Lancman, F. Pruchnik, *J. Mol. Catal.*, **29**, 13(1985); H. Pasternak, F. Pruchnik, K. Wajda-Hermanowicz, M. Zuber, *Polish J. Chem.*, **63**, 619(1986); H. Pasternak, F.P. Pruchnik, *Polish J. Chem.*, **66**, 865(1992).
[11] J.L. Bear, H.B. Gray Jr., L. Rainen, I.M. Chang, R. Howard, G. Serio, A.P. Kimball, *Cancer Chemother. Rep.* **59**, 611(1975); R.A. Howard, A.P. Kimball, J.L. Bear, *Cancer Res.*, **39**, 2568(1979).
[12] K.R. Dunbar, J.H. Matonic, V.P. Saharan, C.A. Crawford, G. Christou, *J. Am. Chem. Soc.*, **116**, 2201(1994).
[13] R.G. Hughes, J.L. Bear, A.P. Kimball, *Proc. Am. Assoc. Cancer Res.*, **13**, 120(1972).
[14] P.N. Rao, M.L. Smith, S. Pathak, R.A. Howard, J.L. Bear, *J. Nat. Cancer Inst.*, **64**, 905(1980).
[15] L.M. Hall, R.J. Speer, H.J. Ridgway, *J. Clin. Hematol. Oncol.*, **10**, 25(1980).
[16] E. Tselepi-Kalouli, N. Katsaros, *J. Inorg. Biochem.*, **40**, 95(1990).
[17] L.D. Dale, T.M. Dyson, D.A. Tocher, D.I. Edwards, *Anticancer Drug Res.*, **4**, 295(1989).
[18] S. Zyngier, E. Kimura, R. Najjar, *Braz. J. Med. Biol. Res.*, **22**, 397(1989).
[19] E.M. Reibscheid, S. Zyngier, D.A. Maria, R.J. Mistrone, R.D. Sinnistera, L.G. Couto, R. Najjar, *Brazil. J. Medic. Biol. Res.* **27**, 91(1994); M.S. Nothenberg, G.K. Takeda, J. Najjar, *J.Inorg. Biochem.*, **42**, 217(1991).
[20] L. Trynda, F.P. Pruchnik, *J. Inorg. Biochem.*, **58**, 69(1995).
[21] L. Trynda, F.P. Pruchnik, *J. Inorg. Biochem.*, in press.
[22] F.P. Pruchnik, D. Duœ, *J. Inorg. Biochem.*, **61**, 55(1996);
[23] F.P. Pruchnik, G. Kluczewska, A. Wilczok, U. Mazurek, T. Wilczok, *J. Inorg. Biochem.*, in press; A. Wilczok, *Biological activity of Rhodium complexes in synchronous culture of Chlorella vulgaris*. Ph. D. Thesis, Silesian Academy of Medicine, Katowice, 1990.
[24] R. J. Bromfield, R. H. Dainty, R. D. Gillard, B. T. Heaton, *Nature*, **223**, 735(1969).

[25] R. D. Gillard, *Kem. Kozlemenyek*, **47**, 107(1977).
[26] N. Farrel, *Transition Metal Complexes as Drugs and Chemotherapeutic Agents*, Kluwer, Dordrecht, 1989.
[27] F. P. Pruchnik, M. Bień, T. Lachowicz, *Metal-Based Drugs*, in press.
[28] G.A. Rempel, P. Legzdins, H. Smith, G. Wilkinson, *Inorg. Synth.*, **13**, 90(1972).
[29] C.R. Wilson, H. Taube, *Inorg. Chem.*, **14**, 405(1975).
[30] B. N. Ames, D. M. Maron, *Mut. Res.*, **113**, 173(1993).
[31] L. Natkaniec, F.P. Pruchnik, *J. Chem. Soc., Dalton Trans.*, 3261(1994).
[32] P. S. Ringrose, in „*The Scientific Basis of Antimicrobial Chemotherapy*", ed. by D. Greenwood and F. O'Grady, Cambridge University Press, Cambridge, 1985, pp 219 - 266.

**Received: September 2, 1996 - Accepted: September 15, 1996 -**
**Received in revised camera-ready format: March 25, 1997**

